# Endothelial Dysfunction in Chronic Inflammatory Diseases

**DOI:** 10.3390/ijms150711324

**Published:** 2014-06-25

**Authors:** Curtis M. Steyers, Francis J. Miller

**Affiliations:** 1 Department of Internal Medicine, University of Iowa, Iowa City, IA 52242, USA; E-Mail: curtis-m-steyers@uiowa.edu; 2 Department of Internal Medicine, Veterans Affair Medical Center, Iowa City, IA 52242, USA

**Keywords:** endothelial dysfunction, endothelium, atherosclerosis, inflammation, inflammatory disease, arthritis

## Abstract

Chronic inflammatory diseases are associated with accelerated atherosclerosis and increased risk of cardiovascular diseases (CVD). As the pathogenesis of atherosclerosis is increasingly recognized as an inflammatory process, similarities between atherosclerosis and systemic inflammatory diseases such as rheumatoid arthritis, inflammatory bowel diseases, lupus, psoriasis, spondyloarthritis and others have become a topic of interest. Endothelial dysfunction represents a key step in the initiation and maintenance of atherosclerosis and may serve as a marker for future risk of cardiovascular events. Patients with chronic inflammatory diseases manifest endothelial dysfunction, often early in the course of the disease. Therefore, mechanisms linking systemic inflammatory diseases and atherosclerosis may be best understood at the level of the endothelium. Multiple factors, including circulating inflammatory cytokines, TNF-α (tumor necrosis factor-α), reactive oxygen species, oxidized LDL (low density lipoprotein), autoantibodies and traditional risk factors directly and indirectly activate endothelial cells, leading to impaired vascular relaxation, increased leukocyte adhesion, increased endothelial permeability and generation of a pro-thrombotic state. Pharmacologic agents directed against TNF-α-mediated inflammation may decrease the risk of endothelial dysfunction and cardiovascular disease in these patients. Understanding the precise mechanisms driving endothelial dysfunction in patients with systemic inflammatory diseases may help elucidate the pathogenesis of atherosclerosis in the general population.

## 1. Introduction

An expanding body of evidence demonstrates that chronic autoimmune inflammatory diseases are associated with accelerated atherosclerosis and increased cardiovascular morbidity and mortality compared to the general population [1,2]. Although rheumatoid arthritis has been most extensively studied, an abundance of data now exists demonstrating excess cardiovascular risk in a multitude of other inflammatory diseases, including systemic lupus erythematosus, the seronegative spondyloarthropathies, psoriasis and inflammatory bowel disease [3,4,5,6,7,8].

Endothelial dysfunction has been postulated to represent an initial step in the pathogenesis of atherosclerosis in the general population [9]. Accordingly, efforts to elucidate unique mechanisms driving increased cardiovascular risk in patients with inflammatory diseases have often focused on the endothelium, which serves as an interface for multiple converging risk factors. In this review, we outline the evidence for and the significance of endothelial dysfunction in several chronic inflammatory diseases. We review the epidemiology and potential mechanisms of endothelial dysfunction in inflammatory diseases, highlighting shared features. Finally, we summarize the available data regarding the efficacy of anti-inflammatory therapies in reducing endothelial dysfunction and potentially mitigating cardiovascular risk.

We queried the PubMed database (NCBI, Bethesda, MD, USA) using the MESH searches for relevant studies using the following search terms in various combinations: rheumatoid arthritis; systemic lupus erythematosus; psoriasis; seronegative spondyloarthritis; inflammatory bowel disease; endothelial function; endothelial dysfunction; endothelial activation; forearm blood flow; flow-mediated vasodilation; cardiovascular disease (CVD); cardiovascular mortality; myocardial infarction; inflammation. Because of the limited number of relevant studies, there were no defined inclusion or exclusion criteria. Studies were screened informally for size and methodological quality. Studies reviewed ranged over the period of 1982–2014, with preference given to more recent data. Systematic reviews and meta-analyses were incorporated when available.

## 2. Endothelial Dysfunction: Definitions and Prognostic Implications

Through its capacity to sense and respond to mechanical and biochemical stimuli, the endothelium plays an active and critical role in the physiologic regulation of vascular tone, cellular adhesion, vascular smooth muscle migration and resistance to thrombosis [9,10]. Endothelial dysfunction—perhaps more appropriately referred to as endothelial activation—refers to its failure to perform these physiologic functions, often as a maladaptive response to pathological stimuli. The phenotypic features of endothelial dysfunction include upregulated expression of cellular adhesion molecules, compromised barrier function leading to increased leukocyte diapedesis, increased vascular smooth muscle tone secondary to impaired processing of vasodilator substances such as nitric oxide and prostacyclin as well as increased production of vasoconstrictor substances including endothelin, and reduced resistance to thrombosis [10]. These processes are thought to represent important steps in the initiation and maintenance of atherosclerosis and have been associated with propensity towards atherothrombosis and cardiovascular complications in advanced disease [9,10,11].

Endothelial dysfunction has emerged as an important surrogate endpoint for cardiovascular events. Its role in initiating the cascade of events leading to atherosclerosis and atherothrombosis may position it well for use as an early indicator of disease at a point that may allow for effective risk factor modification or pharmacologic intervention prior to the development of full-blown atherosclerosis. Furthermore, the endothelium is viewed as an integrator of vascular risk: the mechanisms by which epidemiologically proven cardiovascular risk factors lead to atherosclerosis might be interrogated best at the level of the endothelium, where the processing of these pathogenic signals may converge into one or several common pathways in the genesis of advanced atherosclerosis.

## 3. Assessment of Endothelial Function

Endothelial function can be assessed in humans by assaying its capacity to perform its various physiologic functions, including regulation of vasomotor tone, expression of adhesion molecules and maintenance of an anti-thrombotic microenvironment. In contemporary clinical research, endothelial function is typically assessed by measuring changes in vasomotor tone in response to various stimuli. Methods of measuring vascular reactivity have become the standard largely due to their reproducibility and demonstrated correlation with other measures of atherosclerosis. Quantification of soluble cellular adhesion molecule expression has also been widely performed, although the usefulness of this technique has not been well established. The most common methods are reviewed below.

### 3.1. Forearm Blood-Flow

Quantification of forearm blood-flow (FBF) by venous occlusion plethysmography in response to intra-arterial infusions of vasodilator substances has been historically used to assess vascular reactivity in various patient populations [12]. In this method, endothelial-dependent vasodilation is assayed by intra-brachial infusion of acetylcholine (ACh), an endothelium-dependent vasodilator via induction of endothelial nitric oxide synthase (eNOS) and prostacyclin. The vasodilator response to sodium nitroprusside (SNP), a direct nitric oxide donor and endothelium-independent vasodilator, is also typically assessed in this method. Pure endothelial dysfunction is characterized by impaired vasodilation in response to ACh but intact responsiveness to SNP. FBF has been shown to correlate closely with coronary artery ACh-induced vasodilation [13]. Although reproducible and accurate, FBF measurement is limited by its requirement for arterial cannulation, thereby limiting its repeatability and use in larger cohort studies. 

### 3.2. Flow-Mediated Vasodilation

Flow-mediated vasodilation (FMD) is currently the most widely used method for assessment of vascular reactivity due to its non-invasive nature [12,13,14]. This technique employs ultrasound to measure changes in brachial artery diameter in response to shear stress-induced vasodilation, an endothelium-dependent process. A sphygmomanometer cuff is placed on the patient’s forearm distal to the brachial artery and inflated until all flow ceases. It is then released after a pre-specified period of ischemia, leading to reactive hyperemia secondary to distal microvessel dilation by local factors. The enhanced brachial artery flow is associated with increased shear stress, leading to vasodilation in the presence of a functioning endothelium. This technique has been demonstrated to be endothelium-dependent, as local administration of *N*-mono-methyl-l-arginine (l-NMMA), an inhibitor of NOS, leads to marked reduction in brachial artery dilatation [14]. Studies utilizing FMD also commonly measure brachial artery reactivity in response to oral nitroglycerin, an endothelium-independent vasodilator. The FMD method is often favored over FBF due to its non-invasiveness. It is a technically demanding technique, however, and care must be taken by experienced individuals in order to reduce variability. Regardless of the technical challenges, FMD has been demonstrated to correlate with coronary artery vasoreactivity, markers of subclinical atherosclerosis and future cardiovascular events [13,14,15,16,17].

### 3.3. Microvascular Vasodilation

There has been some concern that assessment of conduit artery function may not accurately reflect endothelial function in the microcirculation. Assessment of endothelium-dependent vasodilation in the cutaneous microcirculation is typically performed by using laser Doppler imaging to measure responses to infusion of vasodilator substances via iontophoresis [18]. Similar to assessment of the larger vessels, ACh is used as the endothelium-dependent vasodilator while SNP is used to assess endothelium-independent mechanisms. These substances are delivered transdermally by application of an electrical field to induce migration of the ionized drug into cutaneous capillaries. Laser Doppler imaging allows for measurement of microvascular perfusion. Various other techniques have been employed to assess microvascular function in tissues other than the skin. Transthoracic echocardiography has been used to assess coronary flow reserve and recently positron emission tomography (PET) has been used to assess myocardial blood flow and coronary flow reserve [19]. These techniques have not yet been widely applied to assessment of endothelial function in patients with chronic inflammatory diseases.

### 3.4. Plasma Biomarkers of Endothelial Dysfunction

Efforts to define plasma biomarkers for endothelial dysfunction have largely focused on soluble intercellular adhesion molecules (CAMs), including intercellular adhesion molecule 1 (ICAM-1), vascular cell adhesion molecule (VCAM-1), E-selectin and others [20,21]. These molecules are typically expressed at the surface of the endothelial cell in response to activation by inflammatory cytokines or other stimuli and bind leukocyte-specific adhesion molecules, leading to increased leukocyte affinity to the endothelial surface and eventually increased transendothelial migration. Although they have been extensively studied, the prognostic value of soluble CAMs remains limited due to poor reproducibility. There is some evidence, however, that elevated ICAM-1 and E-selectin levels are associated with increased risk of incident clinical coronary heart disease [21]. 

Recently, the protein asymmetric dimethylarginine (ADMA), an endogenous eNOS inhibitor, has garnered interest as a potential biomarker for endothelial dysfunction [22]. Plasma levels of ADMA are negatively correlated with NO levels and are elevated in a variety of diseases traditionally associated with cardiovascular risk, including hypertension, dyslipidemia, diabetes mellitus and chronic kidney disease [22]. Elevated plasma ADMA has also been associated with increased risk of cardiovascular events across a range of patient populations [23]. Numerous other molecules, including inflammatory cytokines, regulators of thrombosis and indicators of endothelial damage and repair have been proposed as biomarkers for endothelial dysfunction [24]. The clinical significance of most of these potential biomarkers remains unclear, however.

## 4. Clinical Findings of Major Chronic Inflammatory Diseases Suggest Cardiovascular Risk

Rheumatoid arthritis, systemic lupus erythematosus, the seronegative spondyloarthropathies, psoriasis and inflammatory bowel disease have all been associated clinically with excessive cardiovascular risk [1,2,3,4,5,6,7,8,25]. Over the last several decades, there has been considerable interest in characterizing this excess cardiovascular risk in an attempt to identify potential risk factors and mechanisms responsible for the genesis of atherosclerosis in these populations (Table 1).

**Table 1:** Relative risk of cardiovascular morbidity and mortality.

Disease	CAD Risk (RR or OR)	Cardiovascular Mortality (RR)
Rheumatoid Arthritis	1.5–2.0 [26,27]	1.5 [28]
Systemic Lupus Erythematosus	2.2–2.6 [29,30]	1.7 [31]
Psoriasis (severe)	1.5–7.1 [7,25]	1.1–1.6 [7,25]
Ankylosing Spondylitis	1.9 [32,33]	1.3–2.1 [5]
Inflammatory Bowel Disease	1.2–1.4 [6,34]	1.0 [34,35]

Abbreviations: RR: Relative risk; OR: Odds radio; CAD: Coronary artery disease.

### 4.1. Rheumatoid Arthritis (RA)

It has been known for many years that coronary artery disease is largely responsible for the excess morbidity and mortality in patients with RA. Endothelial dysfunction in RA was first described in a seminal 2002 study demonstrating impaired brachial artery responsiveness to ACh by FBF in patients with early disease [36]. Impaired endothelium-dependent vasodilation has since been repeatedly demonstrated at various stages of disease and across a spectrum of disease activity by several different techniques [37,38,39,40,41]. Microvascular dysfunction has similarly been described in RA, and endothelial-dependent vasodilation in the cutaneous microcirculation has been shown to correlate with disease activity [42,43]. There has been less consistency in the correlation between disease activity and macrovascular function, however. Whereas several studies have demonstrated impaired FMD or FBF in patients with early RA with low disease activity [36,37,39,40], others have failed to show differences in this population [44,45]. Discordance may be related to definitions of “early” and “low” disease activity. A 2012 systematic review of vascular function and morphology in RA included 57 cross-sectional studies and 27 longitudinal studies [46]. The vast majority of these studies reported that endothelium-dependent vasodilation was significantly impaired in patients with RA compared to healthy controls. Studies addressing the correlation between endothelial function and markers of systemic inflammation and disease activity (tender/swollen joint counts, biomarkers of systemic inflammation) were less convincing, however. The authors concluded that the available evidence does not wholly support a correlation between disease activity and macrovascular function [46].

Efforts to characterize endothelial function by measuring soluble plasma biomarkers in patients with rheumatoid arthritis have been largely unsuccessful. Littler *et al.* [47] first described an expression profile of intercellular adhesion molecules in 22 patients with RA. While ICAM-1, ICAM-3, VCAM-1, L-selectin and P-selectin were found to be elevated in sera of patients with RA, only P-selectin correlated with disease activity. Others have identified unique expression profiles in RA patients [48,49,50], although ICAM-1 and P-selectin were also found to be elevated in RA patients in these studies. Several investigators have failed to demonstrate differences in adhesion molecule expression between patients and healthy controls [51]. There is also discordance with regard to the correlation between adhesion molecule expression and markers of disease activity. Plasma levels of ADMA have also been found to be elevated in patients with RA. ADMA levels correlate inversely with FMD and directly with markers of systemic inflammation [52]. In general, the clinical utility of biomarkers for endothelial dysfunction in inflammatory diseases remains unclear. While it appears unlikely that cellular adhesion molecules will serve as important prognostic indicators for CVD, ADMA is more promising. Other biomarkers currently under investigation, such as circulating endothelial progenitor cells, may prove to be useful markers of endothelial dysfunction.

### 4.2. Systemic Lupus Erythematosus (SLE)

The excess burden of CVD in patients with SLE is now well established. Similar to RA, endothelial function has been widely used as a surrogate endpoint for CVD in patients with SLE. Impaired FMD was observed in patients with SLE as early as 2002 [53]. Multiple subsequent studies have validated this observation [54,55,56], including studies interrogating endothelial function in the microcirculation [57]. One study failed to demonstrate differences in FMD between SLE patients and controls, however [58]. Differences in population characteristics may account for this discordance. Importantly, all of these studies excluded patients with known CVD. Taken together, the available evidence strongly supports the presence of impaired endothelium-dependent vasodilation in patients with SLE without documented CVD. 

As with RA, efforts to characterize the expression profile of biomarkers for endothelial dysfunction in patients with SLE have been less successful than vascular reactivity studies. Sfikakis demonstrated increased levels of circulating ICAM-1 in patients with SLE [59]. Tulek and colleagues replicated these results but failed to demonstrate a correlation between ICAM-1 levels and disease activity or markers of systemic inflammation [60]. In contrast, Machold and colleagues failed to demonstrate differences in ICAM-1 levels between SLE patients and healthy controls [61]. Several other groups have attempted to correlate adhesion molecule levels with markers of disease activity. The results have been widely variable, although at least two studies demonstrated a correlation between VCAM-1 levels and disease activity [62,63,64]. Given the heterogeneity between studies and the disparate patterns of results, it is difficult to conclude that patients with SLE exhibit a distinct profile of adhesion molecule expression. There is some weak evidence, however, that during periods of high disease activity and increased systemic inflammation, levels of soluble intercellular adhesion molecules tend to be elevated in patients with SLE. The implications of these findings remain unclear.

### 4.3. The Seronegative Spondyloarthropathies and Psoriasis

Much less is known about the cardiovascular risk associated with the seronegative spondyloarthropathies; however, the available evidence suggests that these diseases confer increased risk of cardiovascular morbidity and mortality [4,5]. Similarly, only a handful of authors have assessed endothelial function in these diseases. Findings have been consistent, however. In two studies of patients with ankylosing spondylitis and one study with psoriatic arthritis, FMD was significantly impaired compared to healthy controls [65,66,67]. One study demonstrated that the reduction in FMD correlated significantly with markers of disease activity [65]. 

Less is known about adhesion molecule expression in this population. As with RA and SLE, the available data are discordant. Wendling *et al.* [68] demonstrated that mildly elevated ICAM-1 levels correlated with markers of disease activity (*i.e*., erythrocyte sedimentation rate (ESR), C-reactive protein (CRP), disease severity score) in patients with spondyloarthropathies. Moreover, markedly elevated ICAM-1 levels (>400) were found only in patients with disease. In contrast, Sari and colleagues failed to demonstrate any significant differences in ICAM, VCAM, E-selectin or P-selectin levels in patients with spondyloarthropathies [69]. Plasma levels of ADMA also appear to be elevated in patients with spondyloarthropathies, although attempts to correlate ADMA levels with markers of disease activity or other markers of endothelial dysfunction have been unsuccessful [70,71].

Cutaneous psoriasis, even in the absence of joint disease, has been linked to accelerated atherosclerosis. The evidence for this association is less robust than for RA and SLE, though the available data support a significantly increased risk for cardiovascular events and mortality in patients with severe disease. Patients with mild disease may be spared [7,25]. Accordingly, many groups have sought to characterize endothelial function in patients with cutaneous psoriasis. Although results have been mixed, a 2014 systematic review of 20 studies of endothelial function in psoriasis patients found that FMD was significantly impaired in the majority of studies in which FMD was used [72]. Some data suggest that the likelihood of endothelial dysfunction is correlated with disease severity or disease duration [73]; however, a number of studies of patients with disease classified as “severe” have demonstrated no impairment in FMD [74,75]. Interestingly, increased carotid intima-media thickness, a measure of subclinical atherosclerosis, has been demonstrated repeatedly in patients with psoriasis and has been shown to correlate with FMD [72,73]. Taken together, these data suggest that patients with psoriasis display impaired endothelial-dependent relaxation and that this may correlate with future development of atherosclerosis and cardiovascular events.

### 4.4. Inflammatory Bowel Disease (IBD)

Similar to psoriasis, there has been recent interest in exploring the cardiovascular implications of inflammatory bowel diseases (IBD), Crohn’s disease and ulcerative colitis (UC). While individual studies have demonstrated an increased risk of cardiovascular events in patients with IBD, mortality risk is less clear. A 2014 meta-analysis of 9 studies demonstrated that patients with IBD were at significantly increased risk of ischemic heart disease and stroke [6]. Mortality was not addressed. Multiple other groups, including Dorn and colleagues in a 2007 meta-analysis of 11 studies, have failed to demonstrate increased risk of cardiovascular mortality in patients with IBD [35]. While there is ample evidence of accelerated atherosclerosis in this population, it remains unclear why these patients are spared from an associated mortality risk. 

In 2003 Hatoum and colleagues demonstrated that the intestinal microvessels in patients with IBD show significantly impaired endothelium-dependent vasodilation compared to vessels from healthy control patients [76]. In addition, the microcirculation in uninvolved segments of bowel remained unaffected, indicating that the mechanism of local endothelial dysfunction may not be related to systemic inflammation. This led to an exploration of generalized endothelial function in patients with IBD. Kocaman and colleagues first demonstrated that generalized endothelial dysfunction is a feature of ulcerative colitis [77]. FMD of the brachial artery was significantly impaired in ulcerative colitis patients, and disease severity was an important determinant of the degree of impairment. Multiple subsequent studies have supported the observation that FMD of the brachial artery is impaired in patients with both UC and IBD, lending credence to the hypothesis that generalized endothelial dysfunction is a feature of IBD, not a tissue-specific phenomenon limited to the GI tract [78,79].

## 5. Animal Models

Multiple models of inflammatory arthritis have been described for the study of rheumatoid arthritis and other inflammatory joint diseases in animals. The collagen-induced arthritis (CIA) model in mice is the most widely-used animal model for the study of RA. In this model, intradermal inoculation of mice with type II collagen and Complete Freund’s Adjuvant (CFA) generates an intense inflammatory polyarthritis immunologically similar to RA [80]. He and colleagues recently demonstrated that CIA mice have significantly impaired endothelium-dependent vasodilation compared to control mice [81]. In a separate study, aortic endothelium isolated from CIA mice was shown to over-express VCAM-1 [82]. Rats with a similar adjuvant-induced arthritis also exhibit both an inflammatory polyarthritis and impaired endothelium-dependent vasodilation [83,84]. A collagen-induced arthritis model was recently described in sheep. Sheep with inflammatory arthritis demonstrated marked impairment in endothelium-dependent vasodilation in the coronary and digital arteries, whereas endothelium-independent vasodilation was intact [85]. Taken together, there is convincing evidence that animal models of inflammatory arthritis exhibit generalized endothelial dysfunction that may provide insight into the mechanisms linking inflammatory diseases and endothelial dysfunction in humans.

## 6. Mechanisms of Endothelial Dysfunction

**Figure 1:**
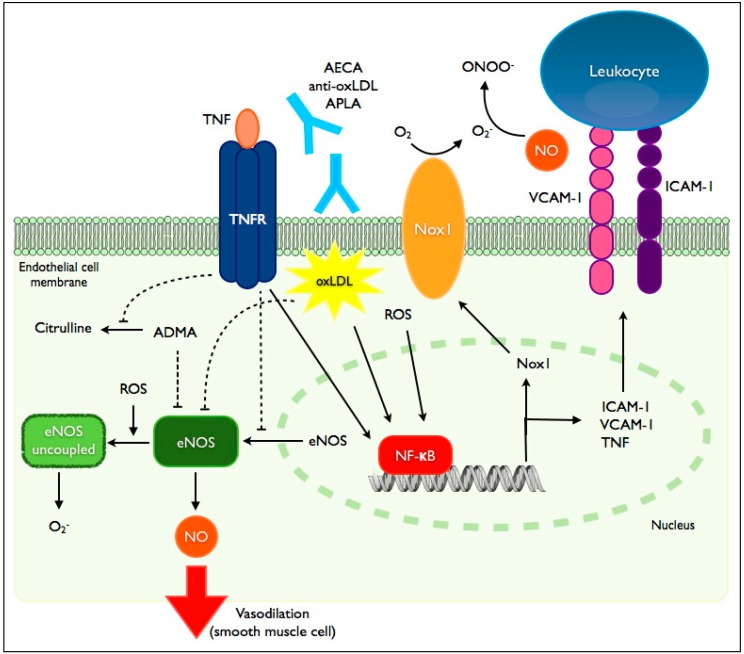
Mediators of endothelial dysfunction in inflammatory diseases. TNF-α (tumor necrosis factor-α) exerts its effects on the endothelium through its receptor, TNFR. Binding of TNFR by TNF-α leads to diminished eNOS (endothelial nitric oxide synthase) protein expression via suppression of promoter activity and destabilization of its mRNA. TNFR suppresses eNOS activity by preventing the degradation of its endogenous inhibitor, ADMA (asymmetric dimethylarginine). TNFR signaling also induces the transcription factor NF-κB leading to enhanced expression of intercellular adhesion molecules (ICAM-1: intercellular adhesion molecule-1; VCAM-1: vascular cell adhesion molecule-1), TNF-α and Nox1 (NADPH-oxidase-1). NF-κB induction is also mediated by oxidized low density lipoprotein (oxLDL), reactive oxygen species (ROS) and binding of various autoantibodies (AECA: anti-endothelial cell antibodies; APLA: antiphospholipid antibodies; anti-oxLDL: anti-oxidized LDL antibodies). eNOS uncoupling, mediated in part by ROS, is associated with reduced NO (nitric oxide) production and enhanced generation of ROS. eNOS activity is also suppressed by oxLDL.

While it is clear that multiple chronic inflammatory diseases are associated with endothelial dysfunction and cardiovascular morbidity, the mechanistic links between inflammatory diseases and CVD have not been fully elucidated. The role of traditional cardiovascular risk factors in patients with RA and SLE has received considerable attention, though traditional factors alone are insufficient to explain the excess burden of CVD in these populations. It seems likely that chronic inflammation, a shared feature of these diseases, is involved in the pathogenesis of accelerated endothelial dysfunction. Several potential mechanisms are explored below (Figure 1).

### 6.1. Tumor Necrosis Factor-α (TNF-α)

The vascular endothelium is known to be a target of TNF-α. On a cellular level, TNF-α induces the expression of genes associated with inflammation, coagulation and proliferation. Decreased nitric oxide (NO) bioavailability appears to be a common and critical step linking TNF-α to endothelial dysfunction. Multiple groups have shown that eNOS protein expression is reduced via TNF-α- induced inhibition of eNOS promoter activity and mRNA destabilization [86,87]. NO availability is also compromised in the presence of TNF-α secondary to impaired degradation of ADMA, an endogenous inhibitor of NOS. Furthermore, TNF-α induces CAM expression on the surface of vascular endothelial cells. This effect is mediated via isoform one of the TNF-receptor (TNFR1). Activation of TNFR1 leads to increased CAM expression via induction of NF-κB [87,88]. NO is also known to be an inhibitor of CAM expression [89]. TNF-α may therefore lead to increased CAM expression by multiple pathways.

The effect of TNF-α on NO availability and subsequent endothelial dysfunction has also been demonstrated *in vivo* in both animal and human models. Intravenous delivery of TNF-α in rats leads to impaired endothelium-dependent vasodilation [90]. Intra-arterial infusion of TNF-α in humans also impairs local endothelium-dependent vasodilation measured by FBF [91]. Non-specific induction of an acute systemic inflammatory response by *Salmonella typhi* vaccination also causes reduced FBF [92]. This effect is mediated by impaired NO bioavailability as demonstrated by rescue of vascular reactivity with the NOS inhibitor l-NNMA (l-N^G^-monomethyl Arginine) [91]. The downstream effects of TNF-α-mediated inflammation are illustrated in an *apoE^−/−^, TNF-α^−/−^* mouse model. Mice deficient in TNF-α develop less atherosclerosis than those with intact TNF-α expression (*i.e*., *apoE^−/−^* single knockout) [93]. This is associated with decreased expression of ICAM-1, VCAM-1 and monocyte chemotactic protein-1 (MCP-1).

**Figure 2:**
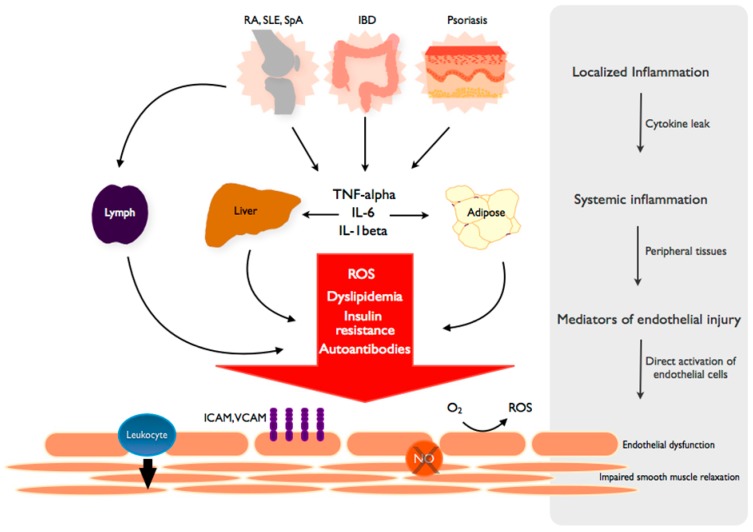
From local inflammation to systemic endothelial dysfunction. TNF and inflammatory cytokines spread from the primary, disease-specific site of local inflammation into the systemic circulation to propagate a systemic inflammatory response. The byproducts of systemic inflammation, including reactive oxygen species (ROS), lipid abnormalities and other metabolic derangements are dependent on peripheral tissues such as the liver and adipose. These mediators elicit independent and complementary effects on the endothelium, leading to a state of endothelial dysfunction characterized by increased adhesion molecule expression (VCAM, ICAM), leukocyte diapedesis, ROS production and decreased NO (nitric oxide)-mediated smooth muscle relaxation and vascular dilation. Autoantibodies are generated in a disease-specific manner and induce similar changes in endothelial function.

It is well known that TNF-α plays a critical role in the inflammation associated with RA, SLE, IBD, psoriasis and spondyloarthritis. This common feature is illustrated by the efficacy of anti-TNF-α agents in many of these diseases. Given the central role of TNF-α in the pathogenesis of many chronic inflammatory diseases and its well-characterized effects on the endothelium as described above, it is reasonable to conclude that increased circulating TNF-α is implicated in the induction of endothelial dysfunction and initiation of atherosclerosis in these diseases (Figure 2). This hypothesis is supported by the beneficial effects of anti-TNF-α agents on endothelial function in patients with chronic inflammatory diseases, as discussed later.

### 6.2. Oxidative Stress

Chronic inflammatory diseases are generally associated with increased oxidative stress. In RA, reactive oxygen species (ROS) levels from peripheral blood neutrophils correlate positively with disease severity and markers of systemic inflammation [94]. Inflammatory cytokines, including TNF-α, are largely responsible for the increased ROS production in these diseases. TNF-α increases activity of the NADPH oxidases (NOX), which catalyze the transfer of electrons onto molecular oxygen to generate superoxide by neutrophils and endothelial cells [87,95]. As discussed previously, the bioavailability of NO is a critical factor in determining vascular reactivity. In addition to its production by NOS and metabolism by ADMA, NO bioavailability is also modulated by ROS. Superoxide rapidly reacts with NO to produce peroxynitrite, thereby decreasing NO availability [96]. The importance of this mechanism is demonstrated by observations that eNOS is paradoxically up-regulated in hypertension and diabetes mellitus, conditions associated with endothelial dysfunction [96]. ROS also contribute to the “uncoupling” of eNOS, leading to enhanced superoxide generation and decreased NO production [97]. Multiple *in vivo* animal models have demonstrated reduced NO bioavailability in the presence of elevated ROS, and reversal of endothelial dysfunction has been achieved via infusion of antioxidants [98].

In addition to downregulating NO bioavailability, superoxide and other ROS are capable of inducing NF-κB, a critical step in transforming endothelial cells into an “activated” state characterized in part by increased surface expression of CAMs [94,99]. As discussed previously, CAM expression by endothelial cells represents a fundamental feature of endothelial dysfunction, leading to enhanced leukocyte affinity and eventually migration into the subendothelial space, key steps in the initiation and maintenance of atherosclerosis. Activation of NF-κB can also stimulate NOX expression, further enhancing ROS production in the endothelium and regenerating the destructive loop of inflammation and oxidative stress [100].

### 6.3. Dyslipidemia

The role of traditional cardiovascular risk factors such as dyslipidemia and insulin resistance in the pathogenesis of endothelial dysfunction and atherosclerosis in patients with chronic inflammatory diseases has received significant attention. Although it has been reported that patients with RA and other rheumatic diseases are more likely to have elevated low-density lipoprotein (LDL) and total cholesterol and reduced high-density lipoprotein (HDL) levels, the data are inconsistent. For example, multiple studies have shown no differences in lipid profiles of patients with RA *versus* healthy controls, whereas others have described a distinct profile of suppressed LDL and HDL in RA patients with more advanced disease (*i.e*., rheumatoid cachexia) [101]. Chronic inflammation structurally alters lipoproteins in ways that are not reflected in standard lipid profiles, however. Inflammation has been shown to modify LDL into small, dense particles that are known to be pro-atherogenic. Indeed, RA patients have elevated plasma levels of small, dense LDL particles [102]. TNF-α also enhances the oxidative modification of LDL by increasing ROS production. In addition, HDL is modified by inflammation. Small HDL particles, known to play a critical role in reverse-cholesterol transport, have been shown to be decreased in patients with RA. The mechanisms by which small HDL is regulated have been extensively reviewed elsewhere [101].

Dyslipidemia is independently associated with endothelial dysfunction. Elevated LDL and total cholesterol are associated with impaired endothelium-dependent vasodilation, whereas elevated HDL levels correlate with improved endothelial function [103]. Impaired endothelial function in dyslipidemic patients may be caused by reduced NO availability. In dyslipidemic patients, NO availability may be impaired by oxidized LDL-mediated reduction in NOS activity or by enhanced metabolism of NO by ADMA [104]. Lipoproteins are also implicated in ROS production via modulation of NOX activity and by contributing to the “uncoupling” of eNOS [105]. In addition to modulation of NO and ROS production, oxidized LDL induces upregulation of CAM expression at the endothelial surface and secretion of TNF-α via induction of NF-kB. These mechanisms are reviewed elsewhere, and additional mechanisms of LDL-mediated endothelial dysfunction have been described in various models [105].

### 6.4. Autoantibodies

Many chronic inflammatory diseases are associated with production of autoantibodies, many of which are instrumental in the pathogenesis of the disease. Similarly, autoantibodies directed against normal endothelial or plasma constituents have been detected and implicated in the pathogenesis of endothelial dysfunction and atherosclerosis in the general population. Anti-endothelial cell antibodies (AECA) directed against a variety of endothelial cell structural proteins have been identified in a number of autoimmune diseases, including SLE [106]. These antibodies have been implicated in the pathogenesis of lupus-associated vasculitis and induce endothelial dysfunction via induction of NF-kB, leading to upregulation of CAMs and inflammatory cytokines [107]. Although these antibodies have been described in SLE and vasculitis, their roles, if any, in the genesis of systemic endothelial dysfunction in SLE and other inflammatory diseases, remain unclear. 

Antibodies directed against oxidized LDL (anti-oxLDL) have been described in patients with and without chronic inflammatory diseases. In SLE, anti-oxLDL antibodies correlate with disease activity and markers of systemic inflammation [108]. Although anti-oxLDL antibodies have been correlated with markers of atherosclerosis in various models, their impact on endothelial cell function remains to be elucidated. There is some evidence that anti-phospholipid antibodies may exhibit cross-reactivity with oxLDL [106]. This would provide a viable mechanism for induction of endothelial dysfunction in patients with SLE and antiphospholipid antibodies. 

Antiphospholipid antibodies (aPLs) are present in the serum of nearly one third of lupus patients. The antiphospholipid antibody syndrome (APS) is characterized by recurrent venous or arterial thrombosis, pregnancy loss and the presence of antiphospholipid antibodies (*i.e*., lupus anticoagulant, anticardiolipin antibody, and anti-B2GPI antibody). Although their contribution to venous and arterial thrombotic events is well known, the relative contribution of aPL to the development of endothelial dysfunction in humans, if any, is currently unclear. The effect of aPL on endothelium-dependent vasodilatation may be reflected in the observation that patients with aPL exhibit impaired FMD and reduced NO bioavailability *versus* healthy controls [109]. aPL have also been shown to enhance CAM expression at the endothelial surface *in vitro* and *in vivo*. Efforts to measure circulating levels of soluble adhesion molecules in patient with APS have been less consistent, however [106]. Further studies are required to clarify whether aPL are responsible for inducing endothelial dysfunction and atherosclerosis in the absence of other complicating risk factors.

## 7. Effects of Pharmacologic Interventions on Endothelial Function

### 7.1. Anti-Inflammatory Therapy

Methotrexate remains the mainstay of therapy for RA and several other rheumatic diseases (Table 2). An inhibitor of folic acid metabolism, methotrexate sharply reduces systemic inflammation and dramatically improves synovitis in patients with inflammatory arthritis. Methotrexate also appears to improve endothelium-dependent vasodilation in patients with RA, although the data are limited [2,110]. Inhibitors of TNF-α have been employed with increasing frequency for patients with a variety of inflammatory diseases, including RA, spondyloarthritis, IBD and psoriasis. 

The critical role of TNF-α in the generation of severe systemic inflammation in these conditions likely explains the effectiveness of these agents. TNF-α may also be largely responsible for the endothelial dysfunction and accelerated atherosclerosis in these patients, making anti-TNF-α agents attractive therapeutic options for preventing CVD in this population. Numerous studies have demonstrated improved endothelium-dependent vasodilation in patients with RA after initiation of anti-TNF-α therapy. This has been demonstrated in a vessel-specific manner by measuring FBF immediately after intra-brachial infusion of infliximab. In this model Cardillo and colleagues demonstrated that the local effect of infliximab on the brachial artery improved brachial artery endothelial function without altering markers of systemic inflammation [111]. Multiple other studies have demonstrated that anti-TNF-α agents improve FMD in RA patients who are refractory to conventional disease modifying anti-rheumatic drugs (DMARD) therapy [112,113,114,115,116,117]. Anti-TNF-α agents also improve endothelium-dependent vasodilation in patients with spondyloarthritis [118,119], cutaneous psoriasis [72,120] and IBD [121], although studies are small and few. Improvement in endothelial function with anti-TNF-α therapy may correlate with improvement in disease activity and markers of systemic inflammation [122]. The duration of the response has been controversial, however. Several studies in RA have shown that anti-TNF-α agents induce a rapid improvement in FMD that is lost after a period of weeks despite effective control of disease activity and systemic inflammation [114,116]. Other studies have demonstrated sustained improvements in endothelial function [115,123]. Factors contributing to differences in duration of response remain unclear.

**Table 2:** Medication and Effect on Endothelial Function.

Medication	Disease(s)	Target	Effect on Endothelial Function
Methotrexate	RA, SpA, Psoriasis	Folic acid metabolism, lymphocyte proliferation, inflammation	Likely beneficial [2,110]
Anti-TNF-α agents	RA, SpA, Psoriasis, IBD	TNF-α-mediated inflammation	Strong evidence for benefit [72,112,113,114,115,116,117,118,119,121]
Corticosteroids	RA, SpA, IBD, SLE	Spectrum of immune and inflammatory responses	Inconclusive [124,125]
Statins	RA, SLE, traditional CVD risk factors	LDL, eNOS, pleiotropic effects on inflammation	Strong evidence for benefit [126,127,128,129]

Abbreviations: RA: rheumatoid arthritis; SpA: spondyloarthritis; IBD: inflammatory bowel disease; SLE: systemic lupus erythematosus; CVD: cardiovascular disease; eNOS: endothelial nitric oxide synthase; LDL: low density lipoprotein.

Anti-TNF-α agents have also been shown to reduce levels of plasma biomarkers of endothelial dysfunction, although results have been inconsistent. Klimiuk *et al.* [130] demonstrated that etanercept administration reduced levels of soluble ICAM-1, VCAM-1 and E-selectin in patients with RA. Gonzalez-Gay and colleagues found reductions only in soluble ICAM-3 and P-selectin after infliximab infusions for patients with RA [131]. Adalimumab therapy in patients with psoriasis has been shown to reduce ICAM-1 levels without affecting other CAMs [120]. These findings are similar to results from studies examining levels of CAMs at baseline across various inflammatory diseases: it has been difficult to discern a consistent profile of CAM expression prior to or in response to disease-modifying therapy. Although CAM expression may be a general marker for systemic inflammation and endothelial dysfunction, its utility in clinical and translational research may be limited.

Corticosteroids have long been used to manage a variety of inflammatory diseases, but their effects on CVD have been controversial. The association between steroids and insulin resistance and obesity has raised concern for increased cardiovascular risk, while their anti-inflammatory effects might mitigate this risk. Studies addressing the association between long-term steroid use in RA and CVD have yielded variable results. A 2011 systematic review of studies of low-dose steroid use in RA found that corticosteroids are generally associated with mildly increased cardiovascular risk [132]. Studies did not reveal an effect of steroids on markers of subclinical atherosclerosis and endothelial function, however. Other observational studies have demonstrated an association between corticosteroid use and lower rates of subclinical atherosclerosis compared to patients not using steroids [133]. Veselinovic and colleagues demonstrated that FMD is higher in RA patients treated with corticosteroids *versus* no therapy [124]. This study conflicts with a randomized prospective study, by Hafstrom, showing that addition of steroids to DMARD therapy does not improve endothelial function in RA patients [125]. These results highlight the difficulty of studying the effects of single-agent steroid therapy on patients with inflammatory disease in the modern era. Measuring the added benefit of steroids in the context of background immune-suppressing therapy is unlikely to reveal significant improvements, even if corticosteroids may have this effect in isolation.

### 7.2. Hydroxymethylglutaryl-CoA Reductase Inhibitors (Statins)

Statins exhibit pleiotropic properties influencing the vasculature that are thought to contribute to their clinical benefit beyond the lipid-lowering effect. Although the mechanisms are incompletely characterized, statins have been shown to improve endothelial dysfunction in patients with traditional cardiovascular risk factors. Potential mechanisms include upregulation of eNOS, leading to enhanced bioavailability of NO and improved vasoreactivity [132]. The use of statins as disease modifying agents and as primary prevention for CVD in patients with chronic inflammatory diseases has also received interest. Statin therapy has been shown to reduce disease severity in patients with RA and has gained attention for use as a disease-modifying agent in other inflammatory diseases [124,125,133,134,135]. Statins have been also been shown to improve endothelium-dependent vasodilation in patients with RA and SLE [126,134,136,137]. This effect appears to correlate positively with measures of systemic inflammation and disease severity [137]. There is current interest in studying the long-term effects of statin therapy on hard cardiovascular endpoints. The Trial of Atorvastatin in Rheumatoid Arthritis was the first randomized controlled trial designed to study the effects of statin therapy in RA patients [136]. At 6 months, statins significantly improved several markers of disease severity and markers of systemic inflammation compared to placebo. Endothelial function was not assessed, however, and the duration of follow-up was not long enough to detect changes in cardiovascular endpoints. Only two studies to date have addressed the effect of statins on cardiovascular events. Sheng and colleagues conducted a population-based cohort study designed to evaluate the effects of statins on lipid levels, cardiovascular events and all-cause mortality in RA and osteoarthritis (OA) patients [138]. Statins similarly reduced lipid levels and were protective of cardiovascular events and mortality in RA and OA patients without prior CVD. There was no protective effect in the secondary prevention setting for either cohort, however. Semb *et al.* [139] demonstrated that statins had a similar effect on cardiovascular events in RA and non-RA patients when used for secondary prevention. Unfortunately, there are no randomized controlled trials addressing the effect of statin therapy on patients with RA.

## 8. Conclusions

Patients with chronic inflammatory diseases are at high risk for cardiovascular morbidity and mortality. In many inflammatory diseases, this heightened risk of CVD is reflected in early endothelial dysfunction as assessed by vasoreactivity studies, even in the absence of detectable atherosclerosis. The endothelium therefore represents an integrator of vascular risk and the study of its dysfunction may help elucidate mechanisms driving accelerated atherosclerosis in these populations.

There is strong evidence that the mechanisms responsible for accelerated atherosclerosis in patients with inflammatory diseases are related to the high-grade inflammation inherent to the primary disease process. The effects of TNF-α and inflammatory cytokines on induction of endothelial dysfunction are well described and are likely to represent key mediators of endothelial dysfunction and atherosclerosis. Furthermore, the numerous studies demonstrating improved endothelial function after anti-TNF-α therapy highlight the importance of these molecules in the pathogenesis of endothelial dysfunction and may lead the way toward advances in pharmacologic prevention of CVD in these populations. Many other mechanisms, including autoantibodies, oxidative stress and interactions with traditional risk factors such as dyslipidemia and insulin resistance are likely to be involved, and further research is required to elucidate the relative importance of these processes. Finally, current strategies to reduce cardiovascular morbidity and mortality are focused on controlling traditional modifiable cardiovascular risk factors and reducing disease activity. The precise mechanisms driving atherosclerosis are likely to vary between different inflammatory diseases, however, and parsing out these subtleties may help identify unique therapeutic targets for each disease.
